# Characterizing Cerebral Imaging and Electroclinical Features of Five Pseudohypoparathyroidism Cases Presenting with Epileptic Seizures

**DOI:** 10.1155/2022/8710989

**Published:** 2022-08-12

**Authors:** Zijuan Qi, Zhensheng Li, Quwen Gao, Li Dong, Jian Lin, Kairun Peng, Wei Xiang, Bingmei Deng

**Affiliations:** ^1^Department of Neurology, General Hospital of Southern Theater Command, No. 111 of Liuhua Road, Yuexiu District, Guangzhou 510010, China; ^2^Department of Neurosurgery, General Hospital of Southern Theater Command, No. 111 of Liuhua Road, Yuexiu District, Guangzhou 510010, China

## Abstract

**Objective:**

To characterize the cerebral imaging and electroclinical features and investigate their etiological contributions to seizures in pseudoparathyroidism (PHP).

**Methods:**

The clinical symptoms, biochemical imaging by magnetic resonance imaging (MRI) and computed tomography (CT) tests, and electroencephalogram (EEG) manifestations of five PHP patients with seizures were retrospectively collected and analyzed.

**Results:**

Physical examination showed an average stature in cases 2~4 and short stature in cases 1 and 5. X-ray tests suggested ectopic calcification in four patients. The seizures in four cases were effectively controlled with antiseizure medicines (ASMs). Cerebral CT scans showed extensive brain calcifications in the bilateral basal ganglia (all five cases), cerebellum (cases 1, 3, and 5), thalamus (case 4), and cerebral cortex. Cerebral MRI showed short T1 signals mainly in the basal ganglia. EEG records revealed focal EEG abnormalities, including abnormal slow waves and epileptiform discharges, mainly over the temporal and frontal lobes. The brain areas with focal EEG abnormalities and calcification did not always coincide.

**Conclusion:**

The seizures in PHP can be focal to bilateral tonic–clonic. ASMs are effective in epilepsy combined with PHP. Intracranial calcification is not a reliable etiological cause of epilepsy in PHP patients.

## 1. Introduction

Pseudohypoparathyroidism (PHP) is one group of rare clinical syndromes characterized by hypocalcemia, hyperphosphatemia, and increased serum concentration of parathyroid hormone (PTH) combined with unique clinical features [1]. The global prevalence and burden of PHP are still unknown, while the estimated prevalence of PHP was reported to be 1.1/100 000 and 1/20 000 in Denmark and the United States, respectively [2]. Due to the rarity of this disease, we also cannot accurately estimate its distribution among people of different age groups. Based on our literature survey on published cohorts and clinical reports (Table S1), PHP can develop at any age from infancy to the elderly. According to the latest consensus statement, PHP diagnosis should be based on multifaceted criteria, including resistance to PTH, ectopic ossification, brachydactyly, and early onset of obesity [1, 3]. Accumulating evidence from scattered clinical cases has outlined the spectrum of clinical manifestations of PHP, which can vary considerably, even among patients carrying the same genetic alteration.

According to the molecular defects and pathogenesis, PHP can be classified into different subtypes, including PHP1a, PHP1b, PHP1c, PHP2, PPHP, and progressive osseous heteroplasia (POH) (Table 1). Upon synthetic PTH administration, PHP1 subtypes show a blunted or no urinary cyclic adenosine monophosphate (cAMP) response, while the PHP2 subtype displays normal cAMP excretion. The main differences in clinical symptoms between PHP1 subtypes are Albright's hereditary osteodystrophy (AHO) features and other hormone resistance besides PTH. Patients with PHP1a/c usually exhibit AHO and multiple hormone resistance, while patients with PHP1b are characterized by PTH resistance but without the phenotype of AHO. In addition to these typical symptoms, many other presentations have been documented in clinical reports, such as neurological symptoms including tetany and epilepsy [4], headache [5], spinal cord compression [6], avascular necrosis [7], nephrocalcinosis [8], cutaneous nodules [9], and dental anomalies [10]. Moreover, extensive cerebral calcification, especially in the basal ganglia areas, has been observed in the imaging results of the reported cases, independent of PHP subtype.

Despite these clinical reports and analyses, whether cerebral calcification is the pathological cause of seizures in PHP remains undetermined. While seizures have been defined to be clinically relevant to PHP and calcification of basal ganglia (BG), the odds for the most common neurological disturbances, including seizures, were similar in patients with and without BG calcification [11]. Some previous works have linked seizures to the decreasing GABAergic inhibition [12], while others have linked calcification to the seizure [13]. To investigate this issue, we retrospectively analyzed the clinical symptoms, biochemical imaging by magnetic resonance imaging (MRI) and computed tomography (CT) tests, and electroencephalogram (EEG) manifestations of 5 PHP patients with seizures to examine these conditions' characteristics and potentially shared etiology.

## 2. Patients and Methods

We retrospectively collected the probands that were clinically diagnosed with PHP from the electronic information system of our hospital from 2016 to 2020. The patients had hypocalcemia with concurrent elevated serum PTH levels for inclusion in this study. Patients were excluded if they had idiopathic or secondary hypoparathyroidism, secondary hyperparathyroidism, severe liver, or kidney function injury and had used medications that could affect calcium/phosphorus metabolism except for calcium or vitamin D.

All patients were questioned about medical and family history, general physical examination, neurological examination, blood biochemical examination, history of birth and development, milestone events, and symptoms and manifestations at the onset of the disease. Basic patient information, including gender, age at onset, history of visits to our hospital, race, and birth location, was collected. Chief complaints including tetany and epilepsy were recorded. Height, weight, and AHO features were noted via physical examination. Previous diagnoses and treatments received at other medical institutions were recorded.

All probands were subjected to imaging tests to evaluate the intracranial calcification using noncontrast CT and/or MRI. Contrast-enhanced CT was conducted according to clinical needs. Cerebral calcification without any other possible causes, such as injury and infection, was considered intracranial calcification. X-ray was used to assess the skeleton calcification of the hands and feet. For patients with suspected seizures, EEG was recorded. Diagnosis of epileptic seizures was conducted by combining self-reported episodes, imaging tests, and EEG records, according to the classification (2017) by the International league against epilepsy (ILAE) [14]. All patients and/or their parents were informed. Patient information and blood samples were collected after patient agreement and under the supervision of the local ethics committee of our hospital.

## 3. Results

**Figure 1:**
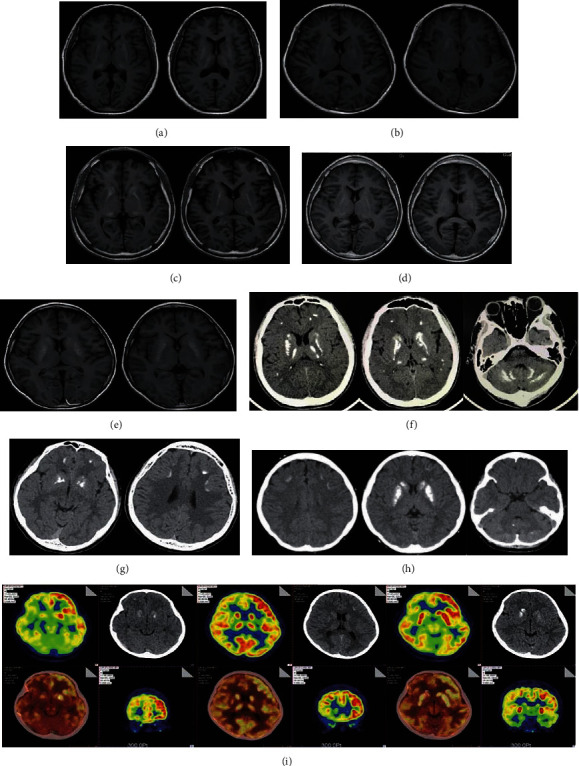
Representative imaging results of all patients. (a)–(e) Representative MR results of cases 1-5. (f)–(h) Representative CT results of cases 3-5. (i) Representative PET-CT results of case 2.

We enrolled a total of 5 PHP patients with recurrent seizures, with the age of onset ranging from 1 to 33 years old (Table 2). All five patients had no relevant medical history of febrile convulsions, encephalitis, and traumatic brain injury, no family history of epilepsy, and no history of substance abuse. Physical examination showed an average stature in cases 2~4 and a short stature in cases 1 and 5; X-ray tests of the feet and hands further revealed nodular shadows with varying degrees in four patients (cases 1~3 and 5), suggesting ectopic calcification (Figure S1). Case 4 did not have a plain film examination. Cerebral CT scans showed extensive brain calcifications in the bilateral basal ganglia (all five cases), cerebellum (cases 1, 3, and 5), thalamus (case 4), and cerebral cortex (cases 2, 4, and 5) (Figure 1; Table 3). Laboratory test results showed reduced serum calcium concentrations (1.50-1.97 mmol/L; normal: 2.15-2.55 mmol/L), raised serum phosphate concentrations (1.09-2.94 mmol/L; normal: 0.97-1.62 mmol/L), and increased serum PTH levels (13.38-41.13 pmol/L; normal: 1.6-6.9 pmol/L) (Table 2). Based on these results, PHP diagnoses were made. For PHP treatment, all these patients have persisted in taking calcitriol.

On admission, all patients reported more than one seizure experience, during which the patients presented with typical symptoms, including uncontrollable jerking movements of the arms and legs and loss of consciousness. Of these five patients, four (cases 2-5; Table 3) were diagnosed with suspected epilepsy by other local medical institutions and had a medication history of antiseizure medicines (ASMs), including valproic acid sodium (VPA; cases 3, 4 and 5), oxcarbazepine (OXC; case 2 and 5), carbamazepine (CBZ; case 5), topiramate (TPM; case 5), and levetiracetam (LEV; case 2). The seizures in three patients (cases 3, 4, and 5) were effectively controlled in the first dosing regimen, while OXC successfully repressed the seizures in case 2 after administration without effect of the LEV.

**Figure 2:**
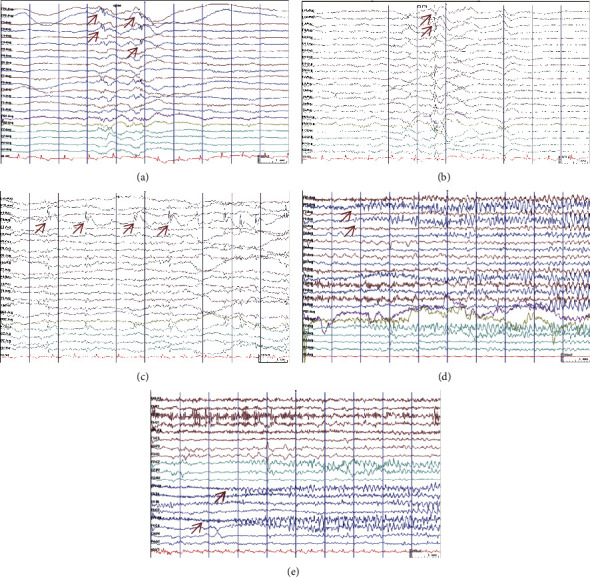
Representative EEG records of all patients. (a, b) Case 3. (c) Case 4. (d, e) Case2. Epileptiform discharges are indicated with arrows in red.

In view of this clinical information, we monitored the brain activities of these patients with interictal EEG (Figures 2(a)–(c)). We also captured the habitual seizure of case 2 (Figures 2(d) and 2(e)). These EEG records showed significant focal EEG abnormalities, including abnormal slow waves and epileptiform discharges, mainly over the temporal and frontal lobes in three patients (cases 2, 3, and 4) (Table 3). We diagnosed “focal to bilateral tonic – clonic seizures” based on these results. We found no obvious interictal epileptiform discharges in cases 1 and 5; thus, the diagnoses of these two patients were based on clinical manifestation.

By comparing the brain areas with abnormal discharges (BADs) with those with calcification (BACs), we found that the BADs in cases 2-4 were only partially overlapped with the BACs. In addition, no common areas between the BADs and BACs were found in cases 1 and 5. Interestingly, the positron emission tomography (PET)-CT scan revealed one brain area in the right frontal lobe of case 2 with low metabolic activity, which was considered associated with the abnormal discharge of this patent. Collectively, our results indicated that intracranial calcification is not a reliable etiological cause of epilepsy in PHP patients.

## 4. Discussion

In this study, we analyzed the clinical features of five PHP patients presented with epilepsy. We speculate that these patients are from different subtypes according to the different characteristics in genetic, clinical, and biochemical features (Table 1). We tracked their medication records and found that most of the ASMs were effective in controlling seizures except for LEV. We compared the brain regions associated with seizures with those with calcification and demonstrated that intracranial calcification is not a reliable etiological cause of epilepsy in PHP.

While all cases in our study presented with hypocalcemia, hyperphosphatemia, and increased serum PTH, only two (cases 1 and 5) were presented with typical AHO features, which suggested a diagnosis of PHP1a/1c. This is because that the PHP1a and PHP1c subtypes can present with identical clinical phenotypes. In contrast, the other three cases were likely to have PHP1b because they presented hypocalcemia, hyperphosphatemia, and increased PTH but without AHO features.

Our study demonstrated that most of the ASMs are possibly effective in controlling seizures of PHP patients, which was consistent with previous study [4]. The ASM regimen has not been included in the clinical consensus of PHP treatment and management [1, 3]. In clinical practice, only approximately 23% of PHP patients were subjected to ASM treatment according to a large cohort study with the Chinese population [4]. Meanwhile, we also noticed some case reports showing that the seizures were controlled after normalization of serum calcium [12, 15]. These collective results suggested that ASM treatment could not be necessary for PHP patients with infrequent seizures. For these patients, the most important thing should be to correct blood calcium levels by taking active vitamin D metabolites, preferentially calcitriol, with or without oral calcium supplementation [1, 3]. Nevertheless, for those PHP patients with frequent seizures, ASM should be considered. For instance, in our study, three out of four PHP patients on ASM treatment have seizure experience as frequently as once one day.

Our study provides clues that intracranial calcification is not a reliable etiological cause of epilepsy in PHP patients. Neurological manifestations are one of the leading clinical symptoms in PHP, including paresthesia, seizures, and tetany [16]. In PHP, intracranial calcification of multiple brain areas, especially BG, has attracted clinical attention for a long time [15, 17, 18]. This ectopic calcification is considered caused by chronic hypocalcemia and associated hyperphosphatemia in PHP, resulting in intracranial calcium deposition due to the elevated levels of the calcium-phosphorus product [3]. Because epileptic seizures can originate in the cortex or subcortical structures [19], whether intracranial calcification is the underlying pathophysiology of seizures in PHP remains undetermined. While BG has been demonstrated to be associated with epilepsy propagation and modulation [20, 21], localizing the epileptogenic focus can help rule out the brain regions where calcification occurs but is not involved in epilepsy. Our study showed that the interictal spikes were multifocal. Notably, we further analyzed the EEG records of the habitual seizures of case 2. We found that the epileptic seizure originated from the right frontal lobe, while the intracranial calcification areas were presented in the bilateral BG, frontal lobes, and cerebellum. Although we cannot completely rule out the contribution of intracranial calcification, our data demonstrated that the exact origin of seizures is not completely consistent with the brain areas with calcification.

PHP represents an unusual form of hormone resistance as the underlying molecular defect is a partial deficiency of the *α* subunit of the stimulatory G protein (Gs*α*), a vital regulator of the cAMP signaling pathway, which is involved in response to multiple hormones, including PTH. PTH resistance is associated with hypocalcemia and hyperphosphatemia. The concentration of calcium ions can affect the activity of synaptic vesicles containing gamma-aminobutyric acid (GABA), which are critical inhibitory neurotransmitters that can reduce neuronal excitation in the brain [22]. Therefore, a reduction of serum calcium has been thought to cause seizures. Nevertheless, the proportion of patients with epilepsy is much smaller than that of patients with hypocalcemia in PHP, indicating that this molecular mechanism is not sufficient to explain the onset of seizures in PHP. Previous studies have demonstrated the critical roles of G protein-coupled receptors (GPCRs) in the central nervous system, in which widely distributed GPCRs can mediate many essential physiological functions by regulating neurotransmission at the synapses [23]. Accumulating evidence has suggested that GPCRs are involved in regulating neuronal excitability associated with epilepsy [24, 25]. The interaction between Gs*α* and GPCRs has been disrupted in most GNAS mutations, a primary molecular determinant of PHP [26]. Therefore, we speculate that the dysfunction of GPCR signaling pathways underlies the mechanism of etiology of seizure of PHP.

Our study tried to shed light on the association between intracranial calcification and epileptic seizures in PHP by combining clinical symptoms, biochemical, and imaging tests. However, there are several limitations. We could not ascertain the molecular subtype by target gene sequencing, usually due to the patients' own choice. We also cannot capture the EEG records of the habitual seizure of all patients, of which only one patient was recorded in our database. For most of the patients, we cannot confirm the exact origin of seizures. Thus, we cannot completely rule out the contribution of brain calcification. In addition, we cannot completely exclude other causes of epilepsy in these cases. For example, the focal PET abnormality in case 2 may be an area of occult focal cortical dysplasia, and PHP could be coincidental. Further studies are required to fill in these gaps.

## Figures and Tables

**Table 1:** Characteristics of PHP subtypes with genetic, clinical, and biochemical features [1, 4, 27].

Subtypes	Genetic feature	Clinical features	Biochemical features
PHP1a OMIM 103580	Maternal LoF of *GNAS*	AHO; hormone resistance (PTH, TSH, GHRH, and gonadotropins)	Low serum Ca; high serum P; high serum PTH
PHP1b OMIM 603233	Deletions in *STX16* or *NESP55*; *GNAS* demethylation	Absence of AHO; hormone resistance (PTH, TSH, GHRH, and gonadotropins)	Low serum Ca; high serum P; high serum PTH
PHP1c OMIM 612462	Mutation in the C-terminal of Gs*α*	AHO; hormone resistance (PTH, TSH, GHRH, and gonadotropins)	Low serum Ca; high serum P; high serum PTH
PHP2 OMIM 203330	Unknown	Absence of AHO; no hormone resistance	Low serum ca; high serum P; high serum PTH
PPHP OMIM 612463	Paternal LoF of *GNAS*	AHO; no hormone resistance	Normal serum Ca, P, and serum PTH
POH OMIM 166350	Paternal LoF of *GNAS*	Deep and invasive heterotopic ossifications	Normal serum Ca, P, and serum PTH

LoF: loss-of-function; PPHP: pseudo-pseudohypoparathyroidism; POH: progressive osseous heteroplasia; AHO: Albright hereditary osteodystrophy; PTH: parathyroid hormone; TSH: thyroid-stimulating hormone; GHRH: growth hormone-releasing hormone; Ca: calcium; P: phosphate.

**Table 2:** Summary of clinical presentations and blood examination.

Case	Sex	Age (year; current/onset)	Seizure type	Seizure frequency and drug treatment	Ca (mmol/L)	P (mmol/L)	PTH (pmol/L)
1	Female	42/33	Secondary	A total of 9 times in 3 years.	1.75	1.09	13.38
2	Male	11/1	Secondary	Y1-3, frequent with unknown exact frequency; Y3-11, a total of 3 times. After Y11, every day ➔ took LEV with a poor effect ➔ took OXC with a good effect, once a year.	1.65	2.72	41.13
3	Male	20/12	Secondary	Y12-15, every day ➔ took VPA with a good effect, no seizures for 2 years ➔ Y15, drug withdrawal; Y18, relapse and took VPA and no seizures till admission.	1.5	1.92	33.74
4	Male	14/11	Secondary	Y11, 2 or 3 times a year ➔ took VPA, and remission, once a year.	1.97	2.94	17.7
5	Female	14/6	Secondary	Y8, every day ➔ took VPA + CBA, seizure free for 2 years, and stop drugs; Y10, relapse and experiencing seizure every day➔ took TPM, and then remission, experiencing seizure twice till on admission.	1.68	2.24	38.2

**Table 3:** Summary of results regarding imaging tests and EEG records.

Case	Cranial MR	Cranial CT	X-ray of limbs	EEG
1	T1 shortening in the bilateral basal ganglia and hypothalamus	Bilateral calcification in basal ganglia and cerebella	Nodular shadows on the first metatarsal bone of both feet	No interictal epileptiform discharges
2^∗^	T1 shortening in the bilateral basal ganglia	Bilateral calcification in basal ganglia and frontal lobes	Nodular shadows on the first finger joint of both hands	Delta waves and spike-slow waves in the right frontal and temporal lobes
3	T1 shortening in the bilateral basal ganglia, frontal lobes, temporal lobes, and cerebella	Bilateral calcification in basal ganglia and cerebella	Nodular shadows on the first metatarsal bone of both feet	Spike-slow waves in the bilateral frontal and temporal lobes and right central region
4	T1 shortening in the bilateral basal ganglia	Bilateral calcification in basal ganglia, frontal and temporal lobes, and thalamus	—	Spike-slow waves in the right frontal lobe, central region, and the frontal midline area
5	T1 shortening in the bilateral basal ganglia and left parietal occipital	Bilateral calcification in basal ganglia, frontal lobes, and cerebella	Nodular shadows on the first metatarsal bone of both feet, the first finger joint of both hands, left planta pedis, and right wrist joint	No interictal epileptiform discharges

^∗^EEG records of habitual seizure, and the others were interictal EEG.

## Data Availability

The imaging data used to support the findings of this study are included in the article.

## Supplementary Materials

Supplementary Materials Table S1: summary of literature survey on published cohorts and clinical reports of PHP patients. We collected a total of 257 PHP patients and the following information from the collected publications: number of patients in each study or case report; patient information including clinical symptoms, age, age of onset, and molecular subtype of PHP; whether the patients were PTH-resistant, hypocalcemia, and hyperphosphatemia; whether epilepsy and tetany occurred at the same time; and EEG records and imaging testing results.
